# Sudden Cardiac Arrest: A Rare Clinical Presentation of Primary Aldosteronism

**DOI:** 10.7759/cureus.42664

**Published:** 2023-07-29

**Authors:** Francisco F Costa Filho, Thomaz A Costa, Alan Furlan, Glenda A de Sa, Madson Q Almeida, Germano E Conceicao-Souza

**Affiliations:** 1 Internal Medicine, Western Michigan University, Kalamazoo, USA; 2 School of Medicine, Universidade Federal do Ceara, Fortaleza, BRA; 3 Cardiology, Hospital Regional de Sao Jose dos Campos, Sao Jose dos Campos, BRA; 4 Endocrinology and Metabology, Department of Internal Medicine, Faculty of Medicine, University of São Paulo (FMUSP), São Paulo, BRA

**Keywords:** adrenal adenoma, ventricular fibrillation, hypokalemia, primary aldosteronism, sudden cardiac arrest

## Abstract

Sudden cardiac arrest (SCA) may be related to reversible causes in up to 50% of cases, such as electrolyte imbalances. Primary aldosteronism (PA) is characterized by excessive autonomic aldosterone production and can present with hypokalemia. We present an uncommon case of a 36-year-old woman who was diagnosed with PA after two episodes of ventricular fibrillation, secondary to severe hypokalemia.

## Introduction

Sudden cardiac arrest (SCA) in young adults is most related to the presence of primary electric diseases and cardiomyopathies, myocarditis, and coronary anomalies [1]. However, reversible causes may account for up to 50% of SCA, and it is imperative to proceed with a causal investigation and an individualized assessment of the necessity for implantable cardioverter-defibrillator (ICD) therapy [1]. Primary aldosteronism (PA) is characterized by excessive autonomic aldosterone production, hypertension, and hypokalemia. In rare cases, significant hypokalemia can lead to life-threatening arrythmias [2]. We present a case of a patient who presented with diffuse weakness, followed by SCA secondary to severe hypokalemia induced by PA.

## Case presentation

**Figure 1:**
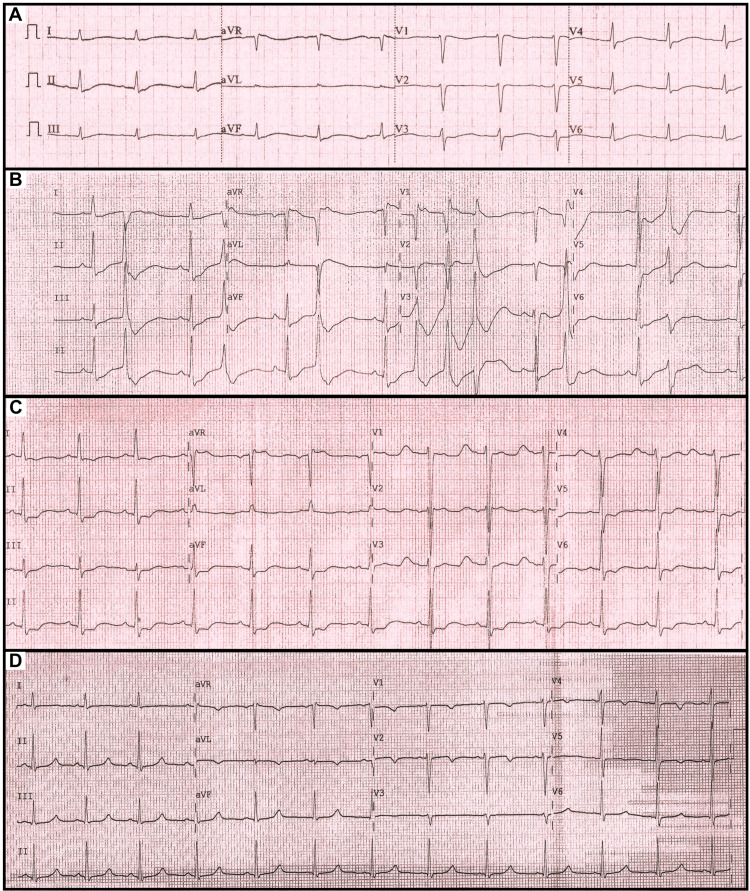
Electrocardiogram (ECG) data over time. (A) ECG post cardiac arrest: sinus rhythm, PR 200ms, QT 600ms, QT-c 671ms. (B) ECG on cardiology unit admission in the presence of severe hypokalemia (K=1.0 mEq/L): sinus rhythm, ST interval depression (V4-V6, DII, DIII, and aVF), and frequent premature ventricular complexes. (C) ECG after 12 hours of K replacement, with serum K of 2.3 mEq/L. (D) ECG on discharge: Sinus rhythm, no ST interval alterations, QT-c 449ms, and serum K of 4.1 mEq/L.

A 36-year-old woman with a past medical history of generalized anxiety disorder (GAD) and poorly controlled hypertension (HTN), using amlodipine 5mg, atenolol 50mg, hydrochlorothiazide 25mg, presented to the urgent care facility due to extremities weakness, paresthesia, nausea, dizziness, and palpitations. Vitals signs revealed a pulse of 93 beats/min (bpm), blood pressure (BP) of 130/80 mmHg, oxygen saturation (SpO2) 98% on room air, and afebrile. Body mass index was 28.6 Kg/m^2^. The patient received symptomatic medications (metoclopramide, dipyrone, and ranitidine) due to concerns of GAD crisis by the first medical provider team. A few minutes after her arrival, patient had sudden loss of consciousness, and was found pulseless on ventricular fibrillation (VF). She was successfully defibrillated and achieved the return of spontaneous circulation (ROSC) after three minutes of cardiopulmonary resuscitation. After the ROSC, she was alert and oriented. Her vital signs were BP 140/90 mmHg, pulse 94 bpm, and SpO2 98%. Her physical exam was positive for frequent extrasystoles and persistence of severe quadriparesis. An electrocardiogram (ECG) obtained after the ROSC demonstrated sinus rhythm and QT-c of 671ms (Figure 1A). The patient was started on intravenous (IV) amiodarone and transferred to our specialized Cardiology Hospital for further management and evaluation of ICD implant. 

On her admission, the patient was found to have severe hypokalemia (K 1.0 mmol/L), as shown in Table 1. ECG revealed new ST depression and frequent premature ventricular complexes (Figure 1B). Shortly after, she developed another SCA in VF during placement of central line in the right internal jugular vein. Again, the patient successfully achieved the ROSC after prompt defibrillation. The central line was placed in the right femoral vein, and the patient was transferred to ICU for continued oral and IV potassium replacement. After 12 hours, the patient improved her muscular strength, and a decreased frequency of extrasystoles was noted (Figure 1C). Spironolactone 100mg daily was added the day after admission.

**Table 1:** Laboratory data over time. * In use of spironolactone 100 mg daily. DST: dexamethasone suppression test. HCO3-: serum bicarbonate. TSH: thyroid stimulating hormone.

Laboratory values	Admission	Day 1	Day 2	Day 12	Reference range (units)
Sodium	144	143	141	135	135-145 (mEq/L)
Potassium	1.0	2.2	3.7	4.1	3.5-5.5 (mEq/L)
Magnesium	2.5	2.5	1.6	1.7	1.17-1.30 (mEq/L)
Ionized Calcium	1.08	1.16	1.02	1.20	1.17-1.30 (mmol/L)
Chloride	85	90	-	91	98-107 (mEq/L)
Urea	50	50	26	-	16-40 (mg/dL)
Creatinine	1.3	1.1	0.8	-	0.6-1.2 (mg/dL)
Hemoglobin	11.8	11.7	-	-	12.0-16.0 (g/dL)
TSH	1.9	-	-	-	0.34-5.6 (UI/mL)
Free T4	1.27	-	-	-	0.89-1.76 (ng/dL)
pH	7.55	7.46	7.46	7.45	7.35-7.45
HCO3^-^	20	21	21	22	22-26 (mEq/L)
Renin	-	-	-	1.4 *	< 4.0 (μUI/mL)
Aldosterone				45.1	< 10.0 (ng/dL)
24-Hours Urine Cortisol	-	-	-	134.6	58-103 (μg/24h)
Serum Cortisol	13.8	-	-	7.26	5.4-25.0 (μg/dL)
Morning Serum Cortisol Post-DST	-	-	-	2.2	< 1.8 (μg/dL)

A work-up to investigate the etiology of hypokalemia was set up. On the review of past medical history, patient confirmed six-year history of hypertension, diagnosed during her last pregnancy. The patient added later that during the previous eight months she had four previous episodes of intense weakness, palpitations, and inability to walk that lasted about two days and had spontaneous resolution. Therefore, a hypothesis of PA was raised.

**Figure 2:**
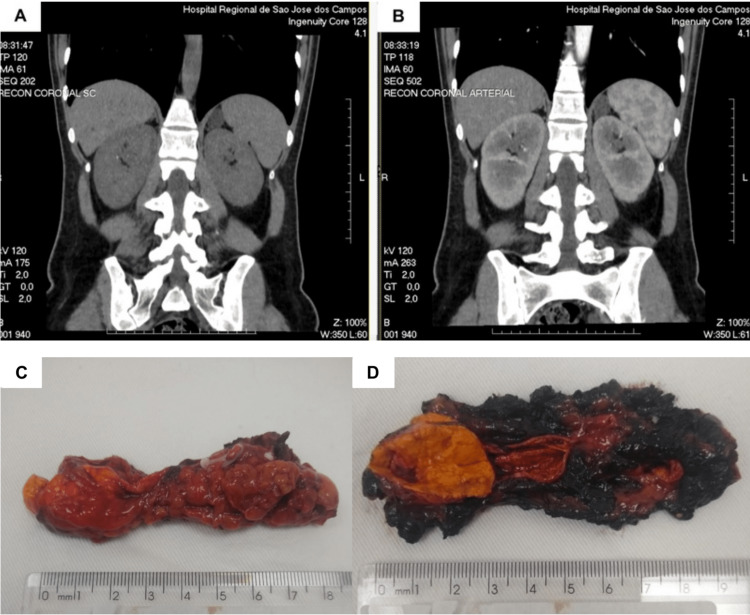
Imaging studies and surgical specimen abdominal computerized tomography scan of abdomen and pelvis revealing a 2.2 x 1.7 cm nodule lesion in the left adrenal gland: (A) Non-contrasted exam and (B) Contrasted exam; (C) Left adrenal gland measuring 8.9 x 3.4 x 1.1 cm presenting yellow/orange solid nodule measuring 2.9 x 2.5 x 1.0 cm; (D) Transverse cut of the surgical specimen.

The results of laboratory test are shown in Table 1. Plasma aldosterone level was elevated at 45.1 ng/dL, and renin concentration was low at 1.4 UI/mL. Abdominal CT revealed a 2.2 x 1.7 cm nodule lesion in the left adrenal gland (Figures 2A, 2B), therefore Conn’s syndrome was diagnosed. After four days of receiving supplemental potassium and spironolactone, she became asymptomatic, with a normal ECG (Figure 1D). Due to her clinical improvement and stability, the cardiology team decided not to proceed with ICD implantation. As the patient presented with symptoms compatible with hyperaldosteronism, along with an aldosterone renin ratio (ARR) higher than 30, and a unilateral left adrenal nodule, she was referred to surgery evaluation. The patient underwent laparoscopic left adrenalectomy, which revealed an adenoma of the adrenal cortex with a predominance of clear cells (Figures 2C, 2D). In a 10-month follow-up appointment, the patient was asymptomatic, free from anti-hypertensive medication, and her BP and potassium levels were normal. 

## Discussion

This case illustrates the uncommon case of a young female with poorly controlled HTN and PA diagnosis after two episodes of SCA secondary to severe hypokalemia, with successful ROSC. Once described as a rare disease, PA diagnosis has increased prevalence mainly due to wide screening in hypertensive patients [3]. In these, the mean prevalence is 5.5%, progressively increasing from 4.2% in patients with stage 1 HTN up to 16.4% in those with stage 3 HTN [3,4].

Hypokalemia, previously described as a classic alteration, is only seen in 9% to 37% of the patients, but might cause life-threatening arrhythmic events [1,2]. The main EKG changes associated with hypokalemia include decreased T wave amplitude, ST-segment depression, T wave inversion, a prolonged PR interval, and an increased QT-c. These findings are present in almost 80% of patients with potassium levels ≤ 2.7 mEq/L [5]. In addition to electrophysiologic cardiac disturbances, hypokalemia is also related to symptoms such as leg cramps, myalgia, muscular weakness, paresthesia, constipation, and nausea [6]. These symptoms are unspecific and might be misinterpreted as anxiety symptoms.

As demonstrated in this case report, early causal investigation for hypokalemia while treating the electrolyte disturbances is fundamental. The most common causes include excessive thiazide diuretics use, laxatives, diarrhea, and vomiting episodes [6]. Renal loss of potassium also may occur with Type I and Type II renal tubular acidosis. Other rarer etiologies like thyrotoxic periodic paralysis, Andersen-Tawil, Batters, Gilteman syndromes, and familial hypokalemic periodic paralysis should be evaluated if the initial diagnostic workup is unclear [6]. In the presented case, they were ruled out based on physical examination and laboratory results. 

When caring for a young adult patient with poorly controlled HTN and a complaint of weakness, excluding endocrinologic causes is recommended [7]. Current guidelines suggest PA screening in patients with a known high prevalence of this disease: treatment-resistant HTN; HTN associated with spontaneous or diuretic-induced hypokalemia, adrenal incidentaloma, or sleep apnea; and patients with a family history of early onset HTN or cerebrovascular accident, or a family history of primary aldosteronism [2,3]. Due to the higher cardiovascular/renal risk in patients with PA compared to those with essential HTN, early diagnosis is vital to prevent comorbidity and mortality [2,3]. Moreover, surgical removal of unilateral tumors has been shown to cure HTN in 50% to 60% of patients [8].

## Conclusions

Unrecognized primary hyperaldosteronism can cause severe hypokalemia followed by ventricular fibrillation and sudden death. Survivals from SCA secondary to hypokalemia and Cohn’s syndrome who successfully underwent adrenalectomy can safely be managed without an ICD implant.
